# The absence of *N*-acetylglucosamine in wall teichoic acids of *Listeria monocytogenes* modifies biofilm architecture and tolerance to rinsing and cleaning procedures

**DOI:** 10.1371/journal.pone.0190879

**Published:** 2018-01-10

**Authors:** Thomas Brauge, Christine Faille, Irina Sadovskaya, Alain Charbit, Thierry Benezech, Yang Shen, Martin J. Loessner, Jean Romain Bautista, Graziella Midelet-Bourdin

**Affiliations:** 1 ANSES, Laboratory for food safety, Boulogne sur Mer, France; 2 UMR UMET, INRA, CNRS, Université Lille 1, Villeneuve d’Ascq, France; 3 Université du Littoral-Côte d’Opale, Institut Charles Violette EA 7394, USC Anses, Boulogne sur Mer, France; 4 INSERM U1151 - CNRS UMR 8253, Paris, France; 5 Institute of Food, Nutrition and Health, ETH Zurich, Zurich, Switzerland; LSU Health Sciences Center School of Dentistry, UNITED STATES

## Abstract

The wall teichoic acid (WTA) is the major carbohydrate found within the extracellular matrix of the *Listeria monocytogenes* biofilm. We first addressed the frequency of spontaneous mutations in two genes (*lmo2549* and *lmo2550*) responsible for the GlcNAcylation in 93 serotype 1/2a strains that were mainly isolated from seafood industries. We studied the impact of mutations in *lmo2549* or *lmo2550* genes on biofilm formation by using one mutant carrying a natural mutation inactivating the *lmo2550* gene (DSS 1130 BFA2 strain) and two EGD-e mutants that lack respective genes by in-frame deletion of *lmo2549* or *lmo2550* using splicing-by-overlap-extension PCR, followed by allelic exchange mutagenesis. The *lmo2550* gene mutation, occurring in around 50% isolates, caused a decrease in bacterial adhesion to stainless steel compared to wild-type EGD-e strain during the adhesion step. On the other hand, bacterial population weren’t significantly different after 24h-biofilm formation. The biofilm architecture was different between the wild-type strain and the two mutants inactivated for *lmo2549* or *lmo2550* genes respectively with the presence of bacterial micro-colonies for mutants which were not observed in the wild-type EGD-e strain biofilm. These differences might account for the stronger hydrophilic surface exhibited by the mutant cells. Upon a water flow or to a cleaning procedure at a shear stress of 0.16 Pa, the mutant biofilms showed the higher detachment rate compared to wild-type strain. Meanwhile, an increase in the amount of residual viable but non-culturable population on stainless steel was recorded in two mutants. Our data suggests that the GlcNAc residue of WTA played a role in adhesion and biofilm formation of *Listeria monocyctogenes*.

## Introduction

*Listeria monocytogenes* is a Gram-positive, aero-anaerobic facultative and intracellular bacterial pathogen that responsible for almost all cases of human listeriosis and can cause severe illness for risk populations [1]. The incidence of *Listeria* infection between 2005 and 2013 varied from 0.26 to 0.32 cases/ year/ 100,000 populations with a 0.8% decrease in the average annual percent change for the same period [2]. Foodborne illness from *L*. *monocytogenes* is characterized by sporadic cases as well as clustered cases or even outbreaks. *L*. *monocytogenes* strains differ in their epidemic potential and in their ability to cause disease with an international estimation of 23,150 illnesses and 5,463 deaths in 2010 [3]. Among the 13 serotypes, serotype 4b strains caused the majority of human listeriosis outbreaks worldwide while the serotype 1/2a and 1/2b strains were isolated mainly from food products. In several epidemic cases of listeriosis, the persistence strains of *L*. *monocytogenes* were reported in an industrial environment [4]. A factor favoring the persistence of *L*. *monocytogenes* is its capacity to form a biofilm that represents a major origin in cross-contamination of food products [5]. A biofilm is a multicellular complex structure, formed of microorganisms that are attached to a surface and generally embedded in an extracellular matrix. Studies have shown that environmental factors, such as temperature, pH or nutrient conditions have an influence on the *L*. *monocytogenes* biofilm formation [6, 7]. The biofilm extracellular matrix is a mixture of exopolysaccharide, DNA, proteins and others extracellular substances [7]. We have recently identified the major soluble carbohydrates in the serotype 1/2a and 4b biofilm matrix as teichoic acids, structurally identical to the cell wall teichoic acids (WTA) of the corresponding serotype [8]. The role of the WTAs in bacterial contamination to inert surfaces remains unknown. Recently, Piercey, et al. [9] demonstrated that the transposition mutation in the *lmo1080* gene involved in teichoic acid biosynthesis inhibited biofilm formation in a simulated food processing plant at 15°C. D-Alanylation of lipoteichoic acids has been also demonstrated as a factor involved in biofilm formation of *L*. *monocytogenes* [10]. Nonetheless, the contribution of WTAs glycosylation in this process has never been studied. However, the presence of a natural mutation (nonsense mutation) in the *lmo2550* gene resulting in the lack of *N*-acetylglucosamine (GlcNAc) in the WTA has very recently been reported with the DSS 1130 BFA2 strain (serotype 1/2a), isolated from seafood product [8]. Autret, et al. [11] and Eugster, et al. [12] have shown that the GlcNAc substitution of serotype 1/2a WTA had an essential function in the recognition of *L*. *monocytogenes* strains by the bacteriophage endolysin PlyP35 as well as the virulence of the *L*. *monocytogenes* cells. Taking into account the importance of the WTA glycosylation in *L*. *monocytogenes*, we investigated the frequency and function of the GlcNAc substitution of serotype 1/2a WTA in the persistence of *L*. *monocytogenes* isolated from industries. In this paper, we attempted to achieve two main objectives. First, we investigated the frequency of mutation of two glycosyltransferase responsible for the GlcNAc substitution of teichoic acids in 93 *L*. *monocytogenes* serotype 1/2a strains isolated mainly from the seafood industry (environment and food). Secondly, we evaluated the impact of the absence of GlcNAc residue on adhesion, formation of *L*. *monocytogenes* biofilms and their further detachment after mechanical and chemical actions.

## Experimental procedures

### Bacterial strains and culture conditions

The *Listeria monocytogenes s*erotype 1/2a studied strains are listed in the Table 1. Cells were stored in brain—heart infusion (BHI) medium (AES, Combourg, France) supplemented with glycerol (18% v/v) at -20°C. All strains were transferred in Tryptone Soy Broth Yeast extract medium (TSBYe) (Oxoid, Dardilly, France) and incubated for 24 h at 30°C with shaking rotator (200 rpm). This suspension was diluted 10-fold in TSBYe and incubated for 24 h at 30°C. Cultures were washed once with 20 ml of MCDB 202 medium (CryoBioSystem, L’Aigle, France) [13] and lastly resuspended in MCDB 202 medium.

**Table 1:** Occurrence of *lmo2550* gene mutation in *L*. *monocytogenes* serotype 1/2a. Accession numbers of the nucleotide sequence data refer to S1 Table.

No mutation			Mutation					
			Nonsense mutation					
Strain	Sampling nature	Reference^1^	Strain	Sampling nature	Références^1^	Mutation of *lmo2550* gene (948pb)	Mutation of Lmo2550 protein (316aa)	Presence of GlcNAc on the wall teichoic acids
A26 P3	Environment	A	B7681 P1	Environment	A	T326_****_^2^	Leu114Stop	No
A3 P3m	Raw salmon		CS5d	Smoked herring	A			
A31 P1	Environment		C3935 PS	Smoked halibut	A	C166T	Gln56Stop	No
B106 OFT9	Raw salmon		C4049 OT	Raw halibut	A			
B136 P2F	Smoked salmon		C3248 OS	Raw salmon	A	C498A	Cys166Stop	No
B674 P1	Smoked tuna		C5125 O	Smoked salmon	A			
B7202 O1	Smoked salmon		C5583 APL1	Smoked trout	A	C832T and A940G	Gln278Stop	No
B7482 O1	Raw perch		DSS 1130 BFA2	Smoked salmon	B	A757T	Lys253Stop	No
B82 P2	Raw salmon		DSS 765 BA3	Smoked herring	A			
C2806 O	Smoked herring							
C3143 O1	Smoked salmon		Silent mutation					
C3299 O1	Raw salmon		B1166 O1	Raw herring	A	C243T	No mutation	Yes
C3615 PS	Smoked herring		B1169 P1	Raw herring				
C4579 OS	Raw halibut		B131 P2	Environment				
C4627 OS	Raw halibut		B7201 P2	Smoked salmon				
C5086 T1	Environment		C1530 O	Smoked herring				
C5128 P	Smoked salmon		C4839 P1	Smoked salmon				
C5142 DNT	Raw salmon		C5067 PS	Smoked herring				
C5316 DNT4	Smoked herring		CP622 CS1	Smoked herring				
C5391 OT	Smoked herring		CS163 P1	Raw herring				
C5400 PT	Smoked salmon		DSS728 CA1	Smoked salmon				
C58 O1	Smoked salmon		DSS843 CFA2	Smoked herring				
C838 P1	Smoked herring		A32 O3	Environment	A	A246G	No mutation	Yes
CL110 P2T48	Environment		CP614 CS2	Smoked herring				
CL219 S2	Environment		CPL631 AS1	Smoked salmon				
CL229 L1	Environment		C4283 PS	Raw herring	A	T285C	No mutation	Yes
CL297 AS1	Smoked salmon	B	CP627 BFS1	Smoked herring				
CP520 APS1	Smoked salmon	A	DA1139.1 E	Raw salmon				
D125 DNT1	Fillet of sole		B7880 P1	Raw herring	A	T315C	No mutation	Yes
D1355 PT1	Smoked salmon		B8005 P1	Smoked herring				
D2022 PS2	Raw herring		C5070 DNT1	Smoked trout				
DA1103 p1	Environment		C54 P1	Raw herring				
DA146 q	Smoked salmon		C5551 OT	Smoked herring				
DA1559 a2	Raw salmon		CS328 CS1	Raw herring				
DA169.2 a6	Raw salmon		CS566 A1	Environment				
DA185 S	Raw salmon		DA1283 q2	Environment				
DA209 q1	Smoked salmon		DA1617 q2	Environment				
DM B5	Environment							
DPF 234 HG2	Raw herring	B	Missense mutation					
DPF235 HG6	Raw herring	A	C1063 O1	Raw salmon	A	A406T	Thr136Ser	Yes
DSS794 AA1	Smoked herring		DA1421 ic2	Raw salmon				
DSS835 CA1	Smoked herring		DA1477 i4	Raw salmon				
DSS836 CS1	Smoked herring		DA509 i1	Environment				
EGD-e	Outbreak	C	DM K17c	Environment				
			D829 OS2	Raw salmon	A	G602T	Arg201Ile	No
			DA1583.3 a3	Smoked salmon	A	C683T	Pro228Leu	No
			B7678 P1	Environment	A	A940G	Ile314Val	Yes
			C3232 O1	Smoked salmon				
			C3572 OS	Raw salmon				
			C5068 PT1	Smoked herring				
			CS536 PL1	Environment				
			CS504 CA1	Smoked herring				
			DA L1	Sporadic case				
			Knock-out mutation					
			EGD-eΔ*lmo2549*	Knock-out mutant	D	-	-	No
			EGD-eΔ*lmo2550*	Knock-out mutant		-	-	No
			EGD-e Δ*lmo2549*:: pLIV2(*lmo2549*)	Complemented EGD-eΔ*lmo2549* mutant	D	-	-	Yes
			EGD-e Δ*lmo2550*:: pLIV2(*lmo*2550)	Complemented EGD-eΔ*lmo2550* mutant		-	-	Yes

^1^: **A:** This study; **B:** Brauge et *al*., 2016; **C:** Glaser et *al*., 2016; **D:** Eugster et *al*., 2011;

^2^: **** indicate a substitution mutation

### Sequencing *lmo2549* and *lmo2550* genes of *L*. *monocytogenes*

We sequenced two genes, *lmo2549* and *lmo2550*, involved in GlcNAc residue of WTA from *L*. *monocytogenes* serotype 1/2a. Ninety three strains of serotype 1/2a *L*. *monocytogenes* isolated in outbreaks, and in seafood product or environment samples were studied as well as a negative control: EGD-eΔ*lmo2549* and EGD-eΔ*lmo2550* (Table 1). Strains were grown on Tryptone Soya Agar Yeast extract (TSAYe) (Oxoid) at 30°C for 24h. Colonies were removed with an inoculating loop and placed in the PCR mix. The PCR reactions were performed according to the protocol of Brauge, et al. [8]. Briefly, a pair of primers for *lmo2549* gene was used: lmo2549F (5’-GGG AAT GGA GTC ATT TGG TT-3’) and lmo2549R (5’-TGC CGT CAT CTT CCC ATT TA-3’). Four primers for the *lmo2550* gene were used: lmo2550F (5’-CTT AAT TTT ATG TAC TAC AAG AGG A-3’), lmo2550R (5’- GTA TAC CAC GGA ATC TTG TC- 3’), lmo2550F2 (5’- CTG GTT AAA GAA AGC AAC TTC A- 3’) and lmo2550R2 (5’- TCT ACG CAT CTT CTA TCGA G- 3’). The lmo2550F and lmo2550R primers were used in PCR. PCR and were performed in an iCycler thermocycler (Biorad, Marnes La Coquette, France) under the following conditions: 94°C for 3 min, 35 cycles of repetition at 93°C for 45 s follow at 54°C for 45 s, and at 72°C for 1 min, and finally at 72°C for 10 min. PCR products were sequenced by the Genoscreen private company (Lille, France) using the amplification primers for *lmo2449* gene and the amplification primers added of lmo2550F2 and lmo2550R2 for the *lmo2550* gene. Alignments of nucleic acid and amino acid sequences were performed by using Bionumerics software (Applied Maths, Sint-Martens-Latem, Belgium).

### Analyzing the monosaccharide composition of wall teichoic acids

After 24 h incubation period at 30°C in TSBYe medium with shaking rotator (200 tr/min), planktonic cultures were centrifuged at room temperature (10.000 x g, 10 min). The cell pellets were washed three times with 5 ml of sterile distilled water and lyophilized. WTA were depolymerized *in situ* by treatment of lyophilized cells with 48% HF, essentially as described in [14]. Two milligrams of lyophilized cells were treated with 48% hydrofluoric acid (Acros Organics, NJ, USA) for 48 h at 4°C. HF was evaporated at room temperature under a stream of nitrogen, 90 μg of internal standard (*myo*-Inositol) and 2 ml of 4 M trifluoracetic acid were added. The insoluble cell debris were removed by centrifugation, and the supernatant, containing WTA, was hydrolyzed at 110°C for 3 h. Monosaccharides were converted to reduced and acetylated derivatives (alditol acetates) by conventional methods and analyzed by GC-MS on a Trace GC ULTRA system (Thermo Scientific) with a DSQ II MS detector, equipped with a capillary column NMTR-5MS (30 m x 0.25 mm) using a temperature gradient of 170°C (3 min) to 250°C at 5°C/min.

### Cell surface hydrophobicity assay

Hydrophobicity was evaluated using a partitioning method, based on the affinity of spores to the apolar solvent hexadecane (MATH) [15]. Briefly, the bacterial culture resuspended in MCDB 202 medium was incubated at 30°C during 3 h. Three milliliter aliquots of the bacterial suspension (absorbance of 0.5–0.6 at 600 nm) and 500 μL of hexadecane (Sigma-Aldrich, Saint-Quentin Fallavier, France) were added in glass tubes (10 mm in diameter × 75 mm), vortexed for times ranging from 5 s to 180 s and left to settle for 30 min to allow complete separation of the two phases. The Gibbs energy of partitioning between the aqueous and hexadecane phases (ΔGpar) is obtained from the equilibrium constant K (ΔGpar = Log K). The equilibrium constant K was calculated from the Equation K = [6(A0 –Aeq) / Aeq)]. The factor 6 in the equation was used to correct for the different volumes of the saline (3 mL) and hexadecane (0.5 mL) phases [16]. A bacterial cell was considered to be hydrophobic for values >2.0, hydrophilic for values ranging from –1.0 and 2.0 and highly hydrophilic for lower values [17].

### Cell adhesion phase to 48h biofilm formation

Biofilm were grown and harvested as previously described with minor modifications [8]. Slides of AISI 316 L 2B stainless steel of 37 mm x 16 mm (Goodfellow, Lille, France) were washed as described in earlier report [18] and were placed in 60-mm-diameter Petri dishes (Nunc, Roskilde, Denmark). Twelve milliliters of the bacterial suspensions of DSS 1130 BFA2, EGD-eΔ*lmo2549*, EGD-eΔ*lmo2550* mutants and wild type EGD-e strain, adjusted to 10^7^ CFU/ml in MCDB 202 medium were deposited in the Petri dish, and were incubated at 30°C for 3 h, 6 h, 24 h, 30 h or 48 h. To prevent any dehydration of the biofilm during incubation, the Petri dish were placed in a 140-cm-diameter Petri dish (Grosseron, Saint Herblain, France) containing a paper towels soaked with 25 ml of distilled water. After incubation, non-adherent bacteria were removed by pouring 25 ml sterilized saline water over the slides.

### Microscopy observations

The morphology of bacterial cells was observed by transmission electronic microscopy (TEM). Bacterial suspensions were adsorbed onto Formvar-coated grids (15/95, Euromedex, EMS, Hatfield, USA) and examined after negative staining with 1% uranyl acetate (VWR, Fontenay-sous-Bois, France) on a Hitachi H600 electron microscope at an accelerated voltage of 75 kV (BICeL platform, Lille, France). For the microscopy observation of bacterial biofilms (samples at different times: 3 h, 6 h, 24 h, 30 h and 48 h), slides were covered with 1 ml of acridine orange colorant (Thermo Fisher Scientific, Villebon sur Yvette, France) or of LIVE/DEAD^®^ Baclight bacterial viability kit (Invitrogen, Carlsbad, California, USA) for 15 min in the dark and then rinsed. The biofilms were examined in a wet state under an epifluorescence microscope (Imager.Z1, Zeiss, Marly-le-Roi, France) connected to a CCD camera (Axiocam—MRm, Zeiss. The biofilm structures were also observed by scanning electron microscope (SEM). Biofilms were fixed during 20 min in glutaraldehyde 3% (v/v) in 0.02M cacodylate buffer, dehydrated in an ethanol series. Biofilms were then subjected to critical point drying, coated with gold-palladium for 1.5 min and viewed on a SEM (Jeol, JSM-35 CF, Japan). For all TEM, SEM and epifluorescence microscopy pictures, six fields of view from two independent experiments have been carried out.

### Quantification of total, viable and viable-culturable biofilm populations

In order to quantify the biofilm sub-populations, biofilm samples were recovered using cotton swabs. Biofilms were collected by thoroughly rubbing the surfaces with a dry swab which was then submerged in an Eppendorf tube containing 2 ml of sterile distilled water and the surface was rubbed again. The procedure was repeated once with a second swab. Both swabs were broken, placed in the Eppendorf tube and vortexed for 1 min. Each suspension was tested to quantify the total, viable (viable culturable (VC) and viable but nonculturable (VBNC) cells) and VC populations. The total and viable populations were quantified by qPCR. The qPCR technique just can amplify the total population (viable and dead). The propidium monoazide (PMA) coupled with qPCR assay allows quantifying the viable population (VC and VBNC). The PMA can bind and covalently cross-link to DNA upon light-exposure. The dye is cell membrane-impermeable, and thus can be selectively used to modify only exposed DNA from dead cells while leaving DNA from viable cells intact. The cell fraction used to quantify the viable population was first treated with propidium monoazide (PMA) (C21H33C12N6, Biotium, Hayward, USA) at a final concentration of 50 μM. The cell fraction was incubated for 5 min at room temperature in the dark, then subjected to light exposure at 80% during 10 min in Eppendorf tube using a PhAST Blue lamp (GenIUL, Barcelone, Espagne). For qPCR assay, the cell fractions were centrifuged (5 000 x g, 10 min) and DNA was extracted with the DNeasy^®^ Blood & Tissue kit (Qiagen, Hilden, Germany) according to the specifications provided by the supplier (protocol “Purification of Total DNA from Animal Tissues”, using ATL lysis buffer). The DNA concentration was measured with a DS-11 Spectrophotometer from Denovix (Wilmington, NC, USA). DNA purity of the samples was checked regarding the A260nm/A280nm and A260nm/A230nm ratio. For relative quantification of extracted DNA, the *hlyA* gene which encodes a pore-forming toxin listeriolysin O was used as a genetic target. qPCR was performed in a total volume of 25 μl containing 2.5 μl extracted genomic DNA, 12.5 μl of SYBR^®^ Premix Ex Taq^™^ (Perfect Real Time), 1μM of forward primer NovF (5′- TGC AAG TCC TAA GAC GCC A-3′) (Eurobio, Les Ulis, France) [19], 1 μM of reverse primer NovR (5′- CAC TGC ATC TCC GTG GTA TAC TAA -3′) (Eurobio) [19] and 9 μl of nuclease free water (Qiagen, Hilden, Germany). The cycling parameters were: 30 s at 95°C followed by 40 cycles of 15 s at 95°C and 30 s at 57°C and 30 s at 72°C. qPCR and data analysis were performed with a Smart- Cycler II^®^ system (Cepheid, Sunnyvale, USA). Cycle threshold (Cq) values were automatically calculated by the SmartCycler software using the second derivative method. All experiments were performed in the presence of a point range for deferral on a standard curve. Standard curves were generated using serial dilutions of genomic DNA extracted with DNeasy^®^ Blood Tissue kit from *L*. *monocytogenes* planktonic culture in TSBYe and covering the range from 10 to 2. 10^9^ genome equivalents (GE) per ml which was calculated from the DNA concentration and based on a *Listeria* genome of 2.94.10^6^ pb [20]. The Cq obtained from the assay of each dilution was used to plot a standard curve by assigning the corresponding concentration values. Standard curves obtained were y = -3.0889x + 41.908; R^2^ = 0.9958 and y = -3.028x + 40.427 R^2^ = 0.9955. The enumeration of the VC population was performed on TSAYe agar (after 24 h incubation at 30°C) using a spiral plater (Spiral System DS, Interscience, Saint Nom La Bretèche, France).

### Resistance of biofilm to rinsing and cleaning procedure

In order to determine the resistance of biofilm to a rinsing procedure, the contaminated stainless steel slides were rinsed with 25 ml of distilled water and placed in rectangular test tubes connected to a CIP rig as previously described [15] and water was circulated for 10 min at a laminar flow rate of 0.16 Pa (= 300 mL/min). After the flow the slides were rinsed by three successive immersions in 200 ml of distilled water. Each experiment was performed twice and each assay was carried out in triplicate for each experiment. Residual cells (dead, VBNC, VC populations) on stainless steel after application of the water flow were enumerated as described above. In order to determine the resistance of biofilm to a cleaning procedure, the same experiments were carried out with NaOH 0.50% (v/v) solution at room temperature.

### Statistical analysis

All the experiments on biofilm were replicated three times for the study of the adhesion to the formation of the 48-h biofilms and 3 slides were replicated three times for the study of the resistance to detachment of 48-h biofilm. Data were analyzed by general linear model procedures using Statgraphics centurion XVI software (Sigma plus, Paris, France). Analysis of variance (ANOVA) were performed to determine the impact of the *lmo2549* and *lmo2550* gene mutations vs wild strain i) of the adhesion step to *Listeria* biofilm formation, ii) on the resistance to detachment of 48h biofilms.

## Results

### Frequency of GlcNAc deficient *L*. *monocytogenes* strains

Two genes, *lmo2549* and *lmo2550*, were sequenced in 93 *L*. *monocytogenes* serotype 1/2a strains isolated from different seafood environments or outbreaks together with two control mutants that harbor in-frame deletions (EGD-eΔ*lmo2549* and EGD-eΔ*lmo2550*) and their respective complemented strains (Table 1). The 93 serotype 1/2a strains were typed by macrorestriction pattern with the *Apa*I and *Asc*I restriction enzymes. The dendrogram of these isolates was based on the profiles obtained with two restriction enzymes (S1 Fig). Fifty-three pulsotypes were identified and studied to have a representation of the diversity of the *L*. *monocytogenes* serotype 1/2a strains in seafood industries. The sequences of the *lmo2549* (438 bp) and *lmo2550* (948 bp) genes of each strain were compared to those of the wild-type EGD-e strain (serotype 1/2a-GenBank accession number AL591824) (Table 1). The sequences of *lmo2549* gene of the 93 strains were identical to that of wild-type EGD-e strain (data not shown). Conversely, 49 of the 93 strains (53%) exhibited a modification of at least one base in the *lmo2550* gene sequence resulting in nonsense, silent or missense mutations. We identified 9 strains with a nonsense mutation. For the C3935PS strain, belonging to the T6k pulsotype, the cytosine at position 166 was substituted by a thymine (C166T), results in a premature stop codon and truncation of Lmo2550 protein that could explain the absence of a GlcNAc residue in its WTA by GC-MS analysis. The same mutation was also identified in the C4049 OT strain belonging to the T6j pulsotype. The *lmo2550* gene sequence (948 nucleotides in length) revealed three other nucleotide substitutions, leading to nonsense mutation in Lmo2550 protein: for two strains, there were two transversions, either a cytosine to adenine at position 498 (C498A) or an adenine to thymine at position 757 (A757T), and for one strain, there were two transitions: a C832T and A940G. Lastly, the deletion of the thymine in position 326 in the two strains, B7681 P1 and CS5d, led to a stop codon. The absence of GlcNAc residue in the WTAs was confirmed by GC-MS assay for the nine strains with a nonsense mutation. We also identified 26 of the 93 studied strains (28%) with a synonymous nucleotide mutation, without modifying the protein sequence of the Lmo2550 and the presence of GlcNAc was confirmed by GC-MS assay: C243T, A246G, T285C and T315C. Lastly, for 15% of the strains studied, *lmo2550* sequencing revealed four nucleotide substitutions leading to four amino acids changed in Lmo2550 protein: A406T (5 strains), G602T (one strain), C683T (one strain), and A940G (7 strains). Only those missense mutations at amino acid position 201 and 228 (leading to Arg201Ile and Pro228Leu) provoked the inactivation of the protein Lmo2550 thereby preventing the GlcNAc branching on the WTA (lack of the GlcNAc residue). We then tried to relate the presence of mutations in the *lmo2250* gene to the sampling nature, the pulsotype, or the sampling site of the *L*. *monocytogenes* strains, but no direct relationship could be demonstrated. Indeed, several strains with different mutations were isolated from the same sampling site and the same sampling nature over years. In addition, even strains with the same pulsotype profiles could have different *lmo2550* gene sequence. In all strains the presence of rhamnose and ribitol in WTAs was confirmed by GC-MS analysis. As controls, in-frame deletion of *lmo2549* and *lmo2550* genes led to the absence of GlcNAc in the cell WTA. Complementation with parental alleles restored the GlcNAc residue on WTA. These observations demonstrate the occurrence of the deficiency in GlcNAcylation of WTA induced by single point mutations in *lmo2549* and *lmo2550* genes.

### Effect of *lmo2549* and *lmo2550* genes deletion on cell adhesion phase at 48 h-biofilm formation

**Fig 1:**
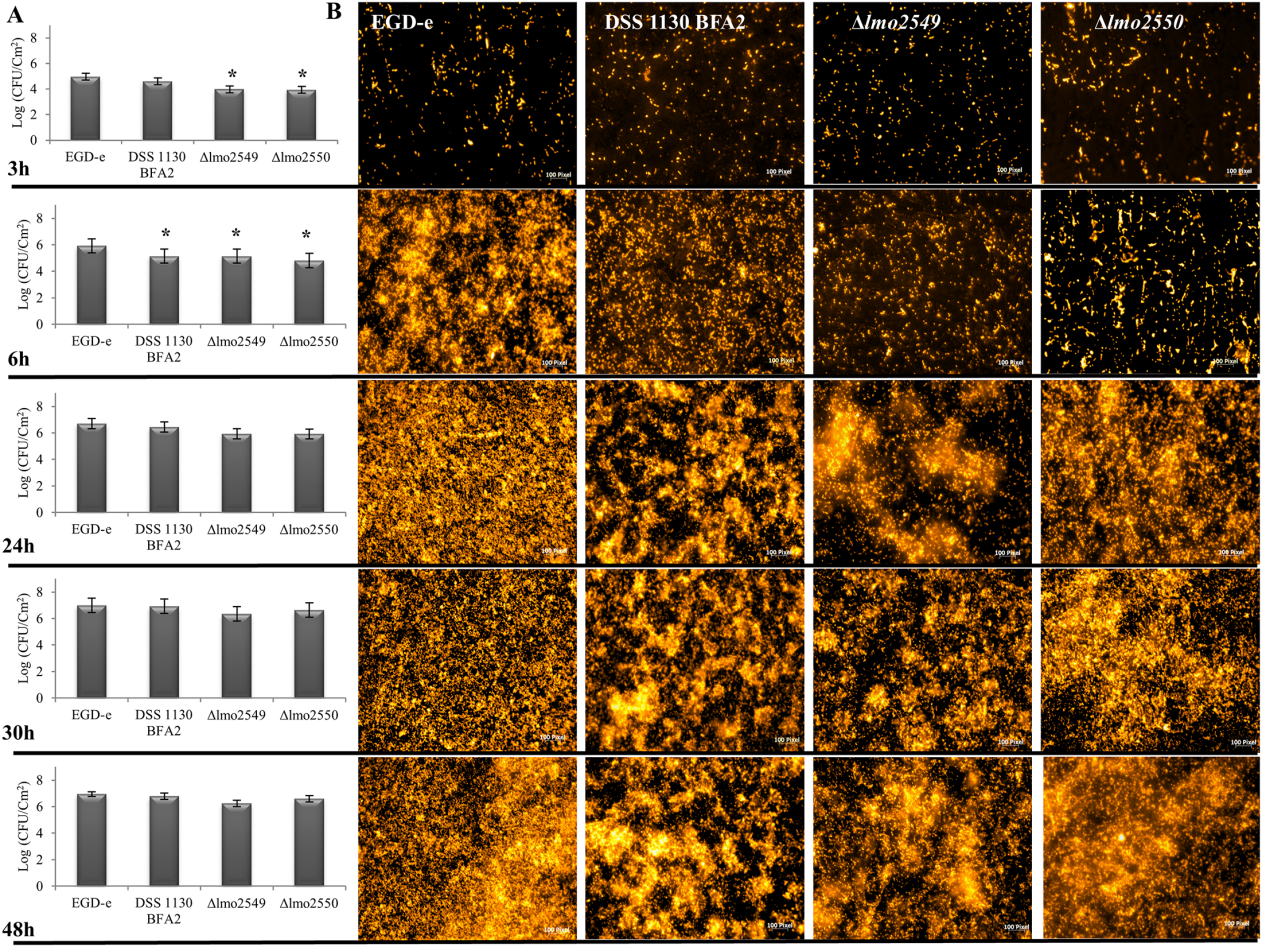
Adhesion to the formation of the 48-h biofilms by the DSS 1130 BFA2, EGD-eΔ*lmo2549* and EGD-eΔ*lmo2550* mutants and the wild-type EGD-e strain grown on stainless steel at 30°C in MCDB202 medium. (A) Enumeration of viable culturable population at t = 3 h; 6 h, 24 h, 30 h and 48 h. The error bars represent the standard deviation (n = 3) and * represent significant difference. (B) Observations by epifluorescence microscopy after staining at acridine orange at t = 3 h, 6 h, 24 h, 30 h and 48 h. The size bar of 100 Pixel indicated 5 μm.

**Fig 2:**
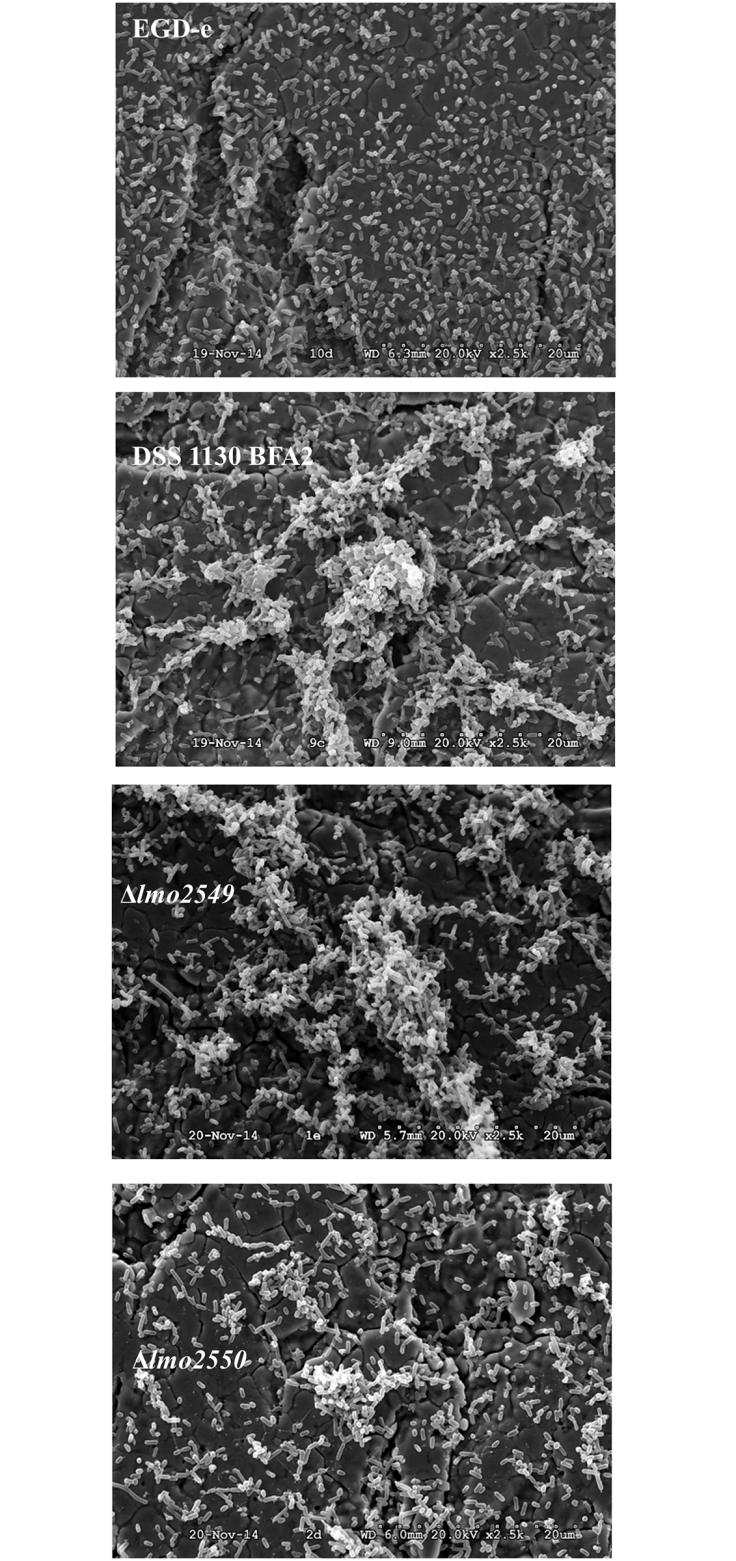
Scanning electron microscopy observations of 48h-biofilm of wild-type EGD-e strain, DSS 1130 BFA2, EGD-eΔ*lmo2549* and EGD-eΔl*mo2550* mutants on stainless steel at 30°C in MCDB202 medium.

We studied the impact of mutations in *lmo2549* or *lmo2550* genes on biofilm formation by using one mutant carrying a natural mutation inactivating the *lmo2550* gene (DSS 1130 BFA2 strain) and two EGD-e mutants that lack respective genes by in-frame deletion of *lmo2549* or *lmo2550* using splicing-by-overlap-extension PCR, followed by allelic exchange mutagenesis. In first step, we validated that all the strains had the similar growth in the MCDB 202 medium during 48h at 30°C (S2 Fig). In order to follow biofilm formation by *L*. *monocytogenes* lacking GlcNAc in WTA, quantification of the viable culturable (VC) population was carried out from the adhesion step (t = 3 h) to the formation of a mature biofilm (t = 48 h) in DSS 1130 BFA2, EGD-eΔ*lmo2549* and EGD-eΔ*lmo2550* mutants vs. wild type EGD-e strain. In parallel, adherent bacteria and biofilms were observed by epifluorescence microscopy after staining by acridine orange colorant (Fig 1). After 3h—adhesion, a greater number of adherent cultivable bacteria was found on surfaces contaminated with the wild-type EGD-e strain and the DSS 1130 BFA2 mutant (around 4.9 log CFU/cm^2^). The deletion in *lmo2549* or *lmo2550* gene resulted in a significant (*P <0*.*05*) decrease in the number of adherent bacteria (decreased around 1 log CFU/cm^2^). Microscopic observations confirmed that the four strains were able to adhere to stainless steel mainly as isolated cells. At t = 6 h, the wild-type EGD-e strain had the highest adherent population with 5.92 log CFU/cm^2^ compared to that of the three mutants (5.14 to 4.81 log CFU/cm^2^). The microscopic observations were coherent compared to the counts because the wild-type strain EGD-e had a higher cell density than the mutants and some micro-colonies were clearly observed. At t = 24 h, 30 h and 48 h, the bacterial population of the biofilms of the wild-type EGD-e strain was similar to the DSS 1130 BFA2, the EGD-eΔ*lmo2549* and the EGD-eΔ*lmo2550* mutants. The wild-type EGD-e strain formed a biofilm with dense cellular carpet-like architecture. It was uniformly distributed across the entire stainless steel surface, with the presence of small cell clusters and extracellular matrix. Larger micro-colonies composed of bacterial cells and extracellular matrix were clearly observed for the biofilm formed by the three mutants. Using scanning electron microscopy (SEM), we then investigated the presence of extracellular matrix in 48 h-biofilm (Fig 2). Within the carpet-like biofilm of the wild-type EGD-e strain extracellular matrix could not be observed. Conversely, bacterial micro-colonies were clearly observed along with isolated cells, and these micro-colonies were found to be composed of bacterial cells more or less embedded in extracellular matrix.

### Cell surface phenotypic characterization

**Fig 3:**
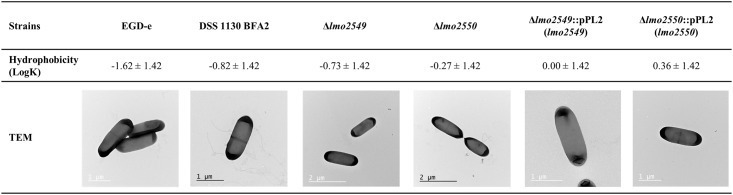
Cell surface characterization of *L*. *monocytogenes* with the hydrophobicity (Log K) (n = 3) and observations by transmission electron microscopy of wild-type EGD-e strain cells, DSS 1130 BFA2, EGD-eΔ*lmo2549*, EGD-eΔ*lmo2550* mutants and the complemented mutants EGD-e Δ*lmo2549*:: pLIV2(*lmo2549*) and EGD-e Δ*lmo2550*:: pLIV2(*lmo2550*).

We estimated the hydrophobic/hydrophilic character of the strains by MATH assay in MCDB 202 medium (Fig 3). All strains had a hydrophilic character. The cell surface of the wild-type EGD-e strain was relatively hydrophobic, as indicated by its high affinity to hexadecane (Log K = -1.62). The hexadecane affinity of the DSS 1130 BFA2, EGD-eΔ*lmo2549* and EGD-eΔ*lmo2550* had a stronger hydrophilic character compared to the wild-type EGD-e strain with a low affinity to hexadecane and a value of Log K of -0.82, -0.73 and -0.27, respectively (*p>0*.*05*). The complemented mutants EGD-e Δ*lmo2549*:: pLIV2(*lmo2549*) and EGD-e Δ*lmo2550*:: pLIV2(*lmo2550*) had also a hydrophilic character. The morphology of the bacteria was investigated by transmission electronic microscopy (TEM) after negative staining (Fig 3). The wild-type EGD-e strain cells were bacilli of 1.63 to 2.13 μm in length and 0.63 to 0.75 μm in width, with the presence of peritrichous flagellae. The morphologies of the DSS 1130 BFA2, EGD-eΔ*lmo2549* and the EGD-eΔ*lmo2550* mutants were similar to that of the wild-type EGD-e strain. Similarly, the complementation of the both deletion restored morphologies similar to that of the wild type strain.

### Resistance to detachment of 48 h-biofilms (mechanical and chemical action)

**Fig 4:**
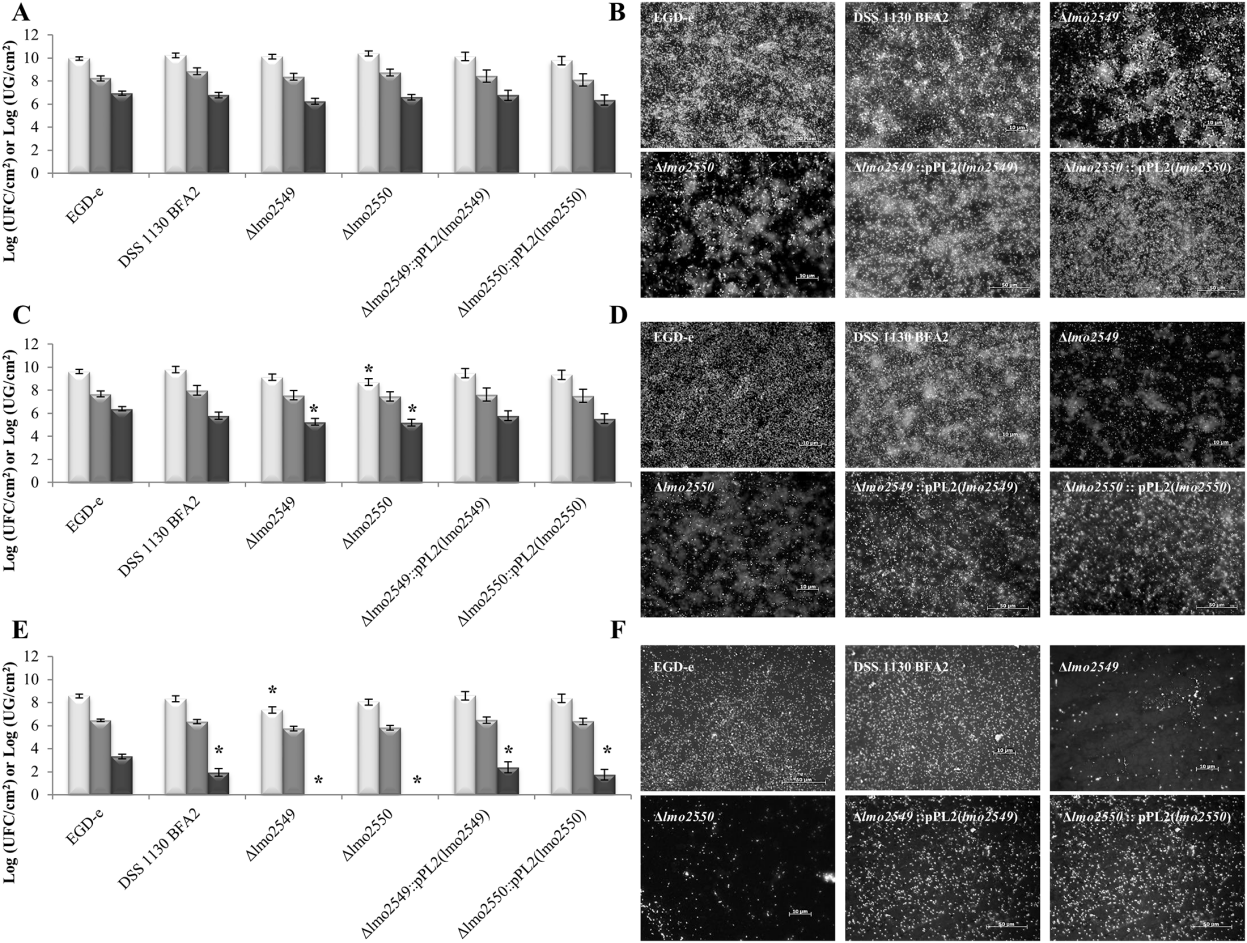
Quantification and microscopic observation of populations from 48h biofilm of *L*. *monocytogenes* DSS 1130 BFA2, EGD-eΔ*lmo2549* and EGD-eΔ*lmo2550* mutants and wild-type EGD-e strain cultivated on stainless steel at 30°C in MCDB202 (A, B), after water flow (C, D), after NaOH flow (E, F). The light gray of histograms indicates total populations, the gray indicates viable populations and the dark gray indicates viable culturable populations. The error bars represent the standard deviation (n = 6) and * represent significant difference.

In order to evaluate their resistance to detachment, biofilms of the six studied strains, produced on stainless steel for 48 h, were subjected to mechanical and/or chemical actions. As described above, the amount of bacteria in 48h biofilm was quantified by qPCR, PMA-qPCR and enumerated on agar (Fig 4A and 4B). The total population quantified by qPCR was similar for the wild-type EGD-e strain,the three mutants and their respective complemented(~10 log CFU/cm^2^) (*P> 0*.*05*). Similar results were observed for the viable population quantified by PMA-qPCR (~8.50 log CFU/cm^2^) and for the VC population enumerated on the agar plates (~6.50 log CFU/cm^2^). These quantifications indicated the distribution of dead, viable but non-culturable (VBNC) and VC populations in the wild-type EGD-e strain. The difference (1.73 log GE/cm^2^) between quantification by qPCR assay and PMA-qPCR assay was an indicative of a dead population. The difference between the quantification by PMA-qPCR assay and the enumeration on the agar plate (1.29 log GE/cm^2^) indicated the presence of VBNC population in the wild-type EGD-e strain biofilm. The VBNC population was significantly higher in DSS 1130 BFA2, EGD-eΔ*lmo2549* and EGD-eΔ*lmo2550* mutant biofilms compared to the wild-type EGD-e strain biofilm (*P < 0*.*05*). On the other hand, the level of dead cell population was similar between the wild-type EGD-e strain biofilm and the DSS 1130 BFA2, EGD-eΔ*lmo2549* and EGD-eΔ*lmo2550* mutant biofilms (*P> 0*.*05*). The complemented mutants EGD-e Δ*lmo2549*:: pLIV2(*lmo2549*) and EGD-e Δ*lmo2550*:: pLIV2(*lmo2550*) behaved like the wild-type strain EGD-e strain. After the application of water rinsing, at 0.16 Pa, on the 48 h-biofilms, we observed that the total populations of the wild-type EGD-e strain, the DSS 1130 BFA2 strain, the EGD-eΔ*lmo2549* and the complemented mutants EGD-e Δ*lmo2549*:: pLIV2(*lmo2549*) and EGD-e Δ*lmo2550*:: pLIV2(*lmo2550*) were not detached contrarily to EGD-e*Δlmo2550* mutant (Fig 4C). For all strains studied, the viable residual populations were in the same proportion, before and after water flow. For the VC population was significantly decreased for the EGD-eΔ*lmo2549* and EGD-eΔ*lmo2550* mutant vs wild-type strain. The VBNC population was significantly higher in three mutant biofilms vs. the wild type EGD-e strain. The microscopic observations of the biofilm showed that the biofilm architecture of the EGD-e strain, the DSS 1130 BFA2 strain and the complemented mutants EGD-e Δ*lmo2549*:: pLIV2(*lmo2549*) and EGD-e Δ*lmo2550*:: pLIV2(*lmo2550*) were not affected by the water rinsing step (Fig 4D). In the EGD-eΔ*lmo2549* and EGD-eΔ*lmo2550* mutants, we observed only few small aggregates still present on the surface after rinsing. This would indicate that cell micro-colonies were slightly adherents. In order to evaluate the resistance to a mechanical shear force coupled with a chemical treatment, the 48 h-biofilms were subjected to a NaOH flow at 0.50% during 10 min at 0.16 Pa. The total residual population on the stainless steel following the passage of the NaOH flow was significantly lower than that of the population in the 48 h-biofilm, but the amount of removed biofilm varied with the strain (between 1.40 log GE/ cm^2^ [wild-type EGD-e strain] and 2.74 log GE/cm^2^ [EGD-eΔ*lmo2549* strain]) (*P <0*.*05*) (Fig 4E). Similar observations were shown with decrease of viable population (between 1.77 log GE/ cm^2^ [wild-type EGD-e strain] and 2.93 log GE/cm^2^ [EGD-eΔ*lmo2550* strain]) (*P <0*.*05*). The significant decrease was also observed for the VC population of the wild-type EGD-e strain (3.61 log CFU/cm^2^), DSS 1130 BFA 2 (4.84 log CFU/cm^2^) and EGD-e Δ*lmo2549*:: pLIV2(*lmo2549*) (4.38 log CFU/cm^2^) and EGD-e Δ*lmo2550*:: pLIV2(*lmo2550*) (4.60 log CFU/cm^2^) (*P <0*.*05*) and no residual VC population was recovered for the EGD-eΔ*lmo2549* and EGD-eΔ*lmo2550* mutants. For all studied strains, a significant increasing of the dead and VBNC populations was recorded after the NaOH flux application. As noted above following a rinsing step, wild-type EGD-e strain was the most resistant to detachment. For all strains, residual isolated cells were observed distributed uniformly on the surface (Fig 4F). The residual cell density was more important for the wild-type EGD-e strain, which confirmed the quantification data. The complemented mutants EGD-e Δ*lmo2549*:: pLIV2(*lmo2549*) and EGD-e Δ*lmo2550*:: pLIV2(*lmo2550*) behaved like the wild-type strain EGD-e strain.

## Discussion

Wall teichoic acids (WTA) of *L*. *monocytogenes* are glycopolymers covalently attached to the peptidoglycan of the cell walls. They are mainly composed of repeated ribitol-phosphate subunits, whose hydroxyl groups can be substituted with a diversity of mono and oligosaccharides. Pronounced diversity in *L*. *monocytogenes* WTA structure is conferred by these glycosidic substitutions. Indeed, WTA of strains of serogroup 1/2 and 3 are substituted by GlcNAc and rhamnose (in the case of serogroup 1/2) substituents, those of serogroup 4 strains are substituted by GlcNAc and, depending on the serotype (4a, 4b, 4ab) [21]. Conversely, there wasn’t substitution for the WTA of the serotype 7. The role of the decorating sugars has been investigated essentially in terms of their contribution to antigenicity [21], resistance to wall-acting antibiotics [22], and virulence [23]. It was shown that the GlcNAc residues of the serotype 1/2 were specifically recognized by the cell wall binding domain of *Listeria* phage endolysin PlyP35 [12], but not of other endolysins, such as Ply118, Ply511, and PlyP40 [24]. Despite the presence of WTA at the cell surface as well as in the biofilm matrix [8], no investigation has been implemented on the possible role of the decorating sugars on the *L*. *monocytogenes* biofilm forming ability to contaminate surfaces. The two glycosyltransferases, Lmo2549 and Lmo2550 are involved in the decoration of GlcNAc of WTA of *L*. *monocytogenes* serotype 1/2a. The *lmo2549* gene has been identified in the wild-type EGD-e strain as encoding a putative glycosyl transferase of 145 amino acids [12], which has 82% similarity with the GtcA protein involved in the decoration of cell WTA of *L*. *monocytogenes* serotype 4b with galactose and glucose [25]. The *lmo2549* gene is flanked by the *lmo2550* gene, which also encodes a glycosyl transferase (315 amino acids). The *lmo2550* gene showed a high degree of similarity with the *csbB* gene encoding for a putative glycosyltransferase in *Bacillus subtilis* [11]. The *lmo2549* and *lmo2550* genes were sequenced from 93 serotype 1/2a strains mainly isolated from seafood industries. The *lmo2549* gene of all strains was 100% identical to that of the wild-type EGD-e strain. In contrast, the *lmo2550* gene was mutated in over 50% of the studied strains resulting in nonsense (10% of the tested strains), silent (28% of the strains) or missense mutations (15% of the strains). Among the nonsense mutants, we confirmed the presence of mutation at nucleotide position 757 of the DSS 1130 BFA2 strain, isolated in smoked salmon, previously described by Brauge, et al. [8]. This mutation was also identified in the strain DSS 765 BA3, isolated from smoked herring in the same factory while in other strains, nonsense mutations resulting from the deletion of other bases, were identified. However, none of these mutations was the result of the deletion previously described by Eugster, et al. [12] in the WSLC 1442 strain. In this strain, the deletion of two nucleotides at position 749 and 750 of the *lmo2550* gene led to a shift of the reading frame and the premature appearance of a stop codon at position 259 of the amino acid sequence. This is the first study describing the important frequency (53%) of mutations in the *lmo2550* gene of the strains isolated in food industries. In their work, Eugster, et al. [12] also constructed two EGD-e strains deleted in *lmo2549* or in *lmo2550* genes and thus confirmed the major role of the GlcNAc residues in the bacteriophage PlyP35 endolysin binding. This resistance to phage of a significant part of the *L*. *monocytogenes* strains isolated in food environments might be of growing concern in the food industries. Indeed, in order to deal with the difficulties in controlling these bacteria in food-processing facilities, the use of bacteriophage in *L*. *monocytogenes* biocontrol has been the subject of many studies [26] essentially because they are extremely host-specific, naturally-occurring and widely-distributed in the environment (and in food) and do not interfere with the resident microflora or starter culture organisms. The WTA being located at the bacterial surface, they probably play a role on their properties, such as the physico-chemical properties and/or the ability to contaminate substrata. This prompted us to investigate the role of the GlcNAc residues of WTA on the adhesion and biofilm formation of *L*. *monocytogenes* on stainless steel. These studies were conducted on the wild-type EGD-e strain and on two mutants obtained by mutagenesis of the *lmo2549* and *lmo2550* genes, and one mutant carrying a natural mutation inactivating the *lmo2550* gene (DSS 1130 BFA2 strain). We first observed that absence of GlcNAc residues resulted in an increase in the hydrophilic character of the cells grown in the MCDB 202 culture medium. This can be explained by the fact that, in comparison with Glc, the GlcNAc is modified on C2, where the alcohol function is replaced by an *N*-acetyl group consisting of an amide function and the backbone acetic acid. This amide function confers a hydrophobic nature of GlcNAc, in agreement with our observations. To date, no comparable studies have been conducted on the impact of TA glycosylation (or D-alanylation) on the hydrophilic character of *L*. *monocytogenes*. Interestingly, this change was accompanied by a decrease in adhesion of the two EGD-e mutants and the spontaneous mutant DSS 1130 BFA 2 strain. Microscopic observations confirmed the low adhesion of the three mutants. This result was expected since the only difference with the wild type is the presence/absence of the GlcNAc residues, which resulted in an decrease hydrophobic character. Indeed, there was broad consensus that hydrophobic cells easily adhere to hydrophobic substrata, such as the stainless steel used throughout this study which is characterized by a water contact angle of 85° [27–29]. On the other hand, these observations have not been confirmed with DSS 1130 BFA2 mutant, which has a natural mutation on *lmo2550* gene, which can be explained by the origin of this mutant and/ or the potential presence of other mutations on gene that promoted the adhesion phase. For incubation times of 24h or more, the presence of GlcNAc residues seemed to play no significant role of the biofilm formation, in terms of both VC populations, despite different adherent bacterial level. Furthermore, the results confirmed similar levels of viable populations within biofilms of the mutant and wild-type strains. This was in agreement with the observations of Brauge, et al. [8] who did not observed any significant difference in the formation of 48 h-biofilm on polystyrene between wild-type EGD-e strain and the DSS 1130 BFA2 spontaneous mutant. In addition, the residual population quantification revealed that mutants lacking GlcNAc substitution have a greater VBNC population compared to the wild-type EGD-e strain. As a reminder, bacterial VBNC forms can be induced by different factors, have a residual metabolic activity, and remain potentially pathogenic. At the favor of certain conditions, they may reactivate, multiply again and become cultivable [30, 31]. The ability of *L*. *monocytogenes* to enter into the VBNC state has previously reported on cells in suspension [32] and in biofilm [33–35]. One can also suggest that the presence of VBNC within biofilm could be the result of a continuous adhesion of VBNC produced in suspending medium. Indeed, Duffy and Dykes [36] found that VBNC *Campylobacter* attached to SSC at a similar level as their counterpart culturable cells. Similarly, Signoretto, et al. [37] showed that the adhesion of the VBNC population of *Enterococcus faecalis* to plankton was an important mechanism for its persistence in aquatic environments. The presence of many VBNC within *L*. *monocytogenes* biofilms may be a great concern in the food industry, mainly because they cannot be detected by traditional culturing methods. Despite the similarities observed in bacterial quantification within biofilms in the presence or absence of GlcNAc residues, the biofilm architecture was clearly affected with the presence of micro-colonies and cell clusters clearly observed by optical microscopy and by SEM while a carpet-like organization was observed for the wild-type EGD-e strain. Similar observations were made in confocal microscopy by Danese, et al. [38] on the mutants of *Escherichia coli* K-12 inactivated in the biosynthesis pathway (a major exopolysaccharide of this bacterium). To our knowledge, there are no similar studies on the possible role of WTA glycosylation on adhesion phase to the biofilm formation for wild-type and mutants of *L*. *monocytogenes* strains. It is therefore the first time that the role of some WTA decorations on the interaction forces between bacteria and substrata was demonstrated probably through their impact on the bacterial surface hydrophilic character. Interestingly, a study on *Bacillus* spores appears to support our finding: hydrophilic *B*. *subtilis* spores were more easily detached in a water flow than hydrophobic *B*. *cereus* spores [39]. We also investigated the possible role of the GlcNAc residues on the resistance to cleaning (both mechanical and chemical actions) of *L*. *monocytogenes* strains belonging to the serotype 1/2a. The mechanical action alone (water flow, wall shear force of 0.16 Pa) failed in removing wild-type EGD-e biofilm, reflecting strong biofilm cohesion and adhesion to substrata. In the food industry, the mechanical action induced when rinsing and cleaning equipment could greatly vary from 0.01 Pa up to more than 20 Pa due to e.g. potential variation of the equipment diameter changes in processing lines. The wall shear conditions tested here (0.16 Pa) fell in actual industrial conditions encountered in e.g. valves and tanks. We demonstrated here that *L*. *monocytogenes* biofilms were highly resistant to cleaning with less than 2-log decrease of the total population whatever the studied strain. Conversely similar fractions of the populations were viable or VBNC before or after the rinsing step indicating that the different biofilm sub-populations were equally sensitive to shear stresses. This resistance to cleaning is even more evident when comparing to data obtained on *Bacillus* spores, known to be highly resistant to cleaning: the use of 0.50% NaOH at 4 Pa, resulted in the removal of less than 99% of adherent *Bacillus* spores [39]. This high resistance might be of concern in the food industry, especially because strains of *L*. *monocytogenes* serotype 1/2 have also been demonstrated to be highly resistant to disinfection procedures [40, 41]. Furthermore, it is widely admitted that stainless steel (used in this study) is more hygienic than other materials used in the food industry, as evidenced by the differences in the resistance to disinfectant observed on *L*. *monocytogenes* biofilms grown on stainless steel, polyester and polyester/polyurethane [42]. Despite this overall trend, both EGD-e mutants were more easily removed from the contaminated surface. As in the case of biofilm detachment with a water flow, one can hypothesize that the differences are in part due the modifications in surface physico-chemistry resulting from the absence of GlcNAc residues. Similar results have been previously reported on *Bacillus subtilis* spores, which became less hydrophilic and more resistant to detachment following the mechanical removal of an external mucous [17]. Elsewhere, within the residual biofilm, a significant fraction of the VBNC was also detached during the cleaning procedure. Lastly, the VC population was strongly decreased but the dead and the VBNC populations were strongly increased, as expected following a treatment with NaOH flow. Indeed, it is known that many pathogens are able to survive environmental stresses such as low nutrient concentration or disinfectants such as chlorine [43] by entering into a VBNC state, going undetected by culture methods. More interesting, this phenomenon was strongly accentuated with the mutants without GlcNAc residue on WTA, but no conclusion may be drawn concerning the detachment of these culturable cells: are they detached during cleaning or are they make VBNC without (or with) further detachment.

In conclusion, this work has established for the first time a role for WTA glycosylation in surface contamination by *L*. *monocytogenes* serotype 1/2. We further showed that this phenotype was widespread in the seafood industry (surfaces and food). These results highlight areas of concern in the food environment that require further study. First, we will now compare the behaviour of environmental mutants to their complemented counterpart to determine the ubiquitous character of the role of the GlcNAc residues on WTA. In parallel, it would be interesting to extend the sequencing of the gene *lmo2550* to the *L*. *monocytogenes* strains from different industry (meat, vegetables or milk) in order to observe whether the strong mutation of the *lmo2550* gene is specific to the seafood industry by the presence of certain specific bacteriophages.

## Supporting information

S1 Fig Dendrogram of obtained using *ApaI* and *AscI* enzymes demonstrating the genetic diversity of *L*. *monocytogenes* isolates. (PDF)

S2 Fig Growth of the wild-type EGD-e strain, DSS 1130 BFA2, EGD-eΔ*lmo2549* and EGD-eΔ*lmo2550* mutants and the complemented mutants EGD-e Δ*lmo2549*:: pLIV2(*lmo2549*) and EGD-e Δ*lmo2550*:: pLIV2(*lmo2550*) at 30°C in MCDB202 medium during 48 h. (PDF)

S1 Table Accession numbers of any nucleotide sequence data. (PDF)
