# Supplementary figures and images for: The absence of N-acetylglucosamine in wall teichoic acids of Listeria monocytogenes modifies biofilm architecture and tolerance to rinsing and cleaning procedures
Source: PLoS One. 2018 Jan 10;13(1):e0190879. doi: 10.1371/journal.pone.0190879 (PMC5761963; doi:10.1371/journal.pone.0190879)

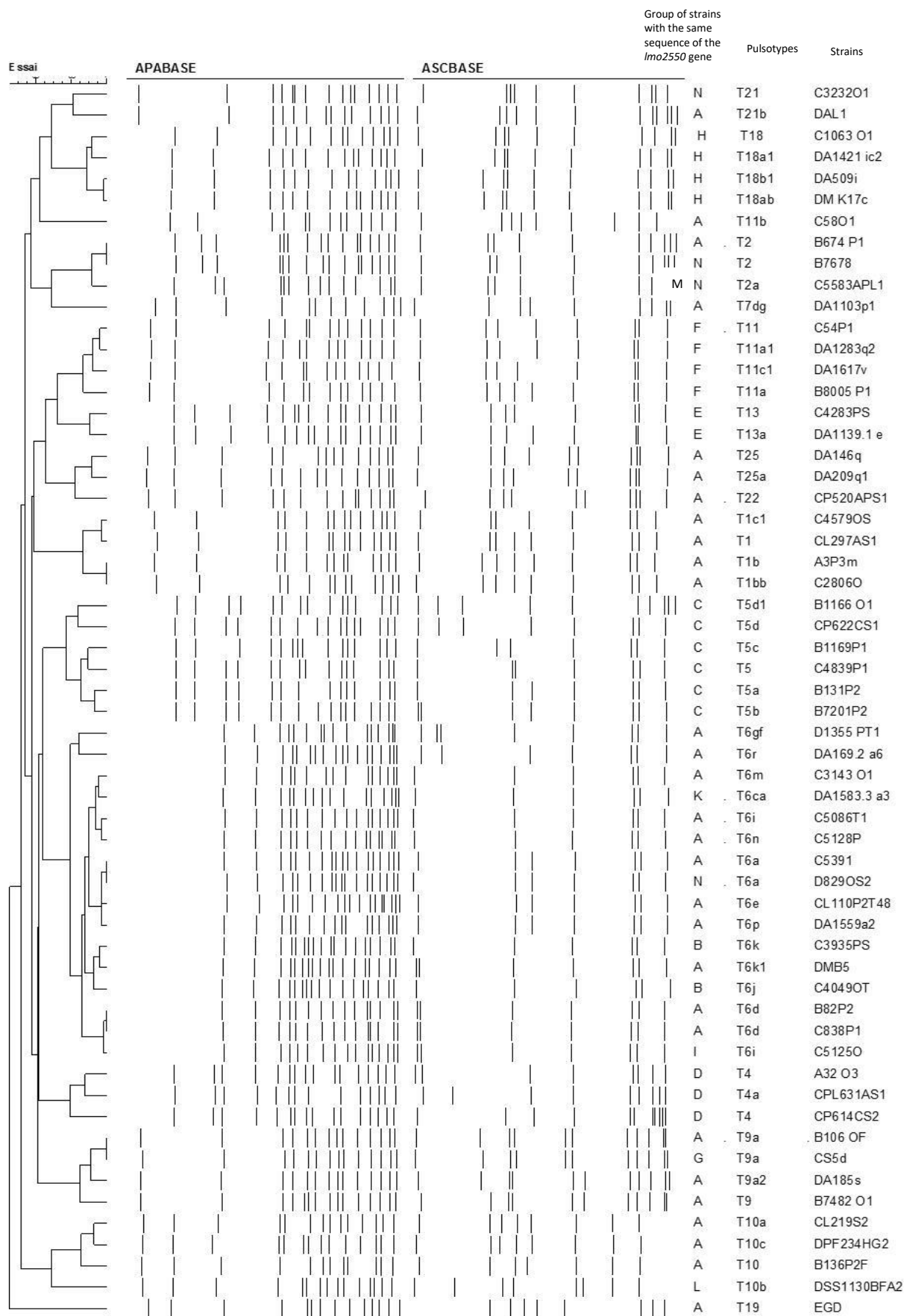

Fig.S1.

Supplement: S1 Fig — (PDF) [file pone.0190879.s001.pdf]

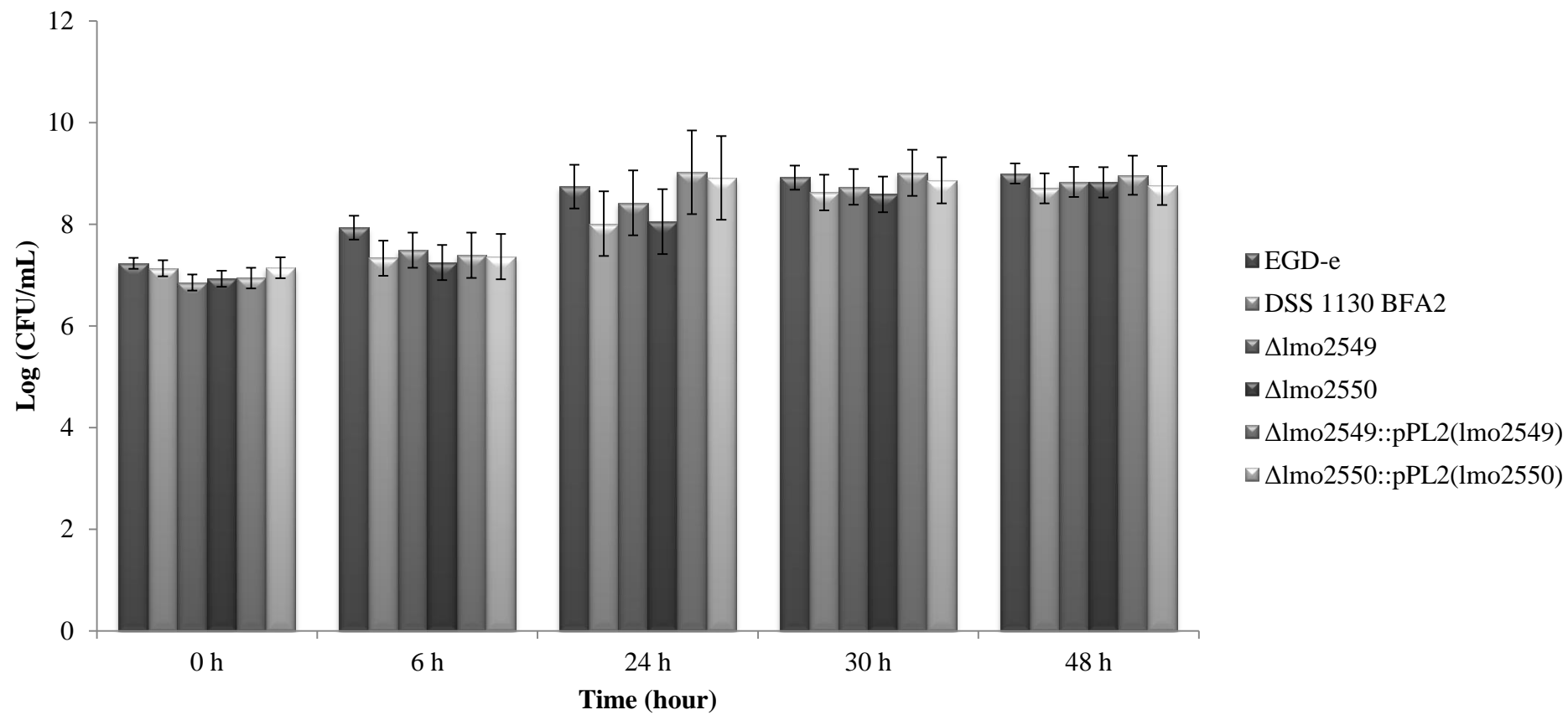

Supplement: S2 Fig — (PDF) [file pone.0190879.s002.pdf]
