# Supplementary material for: The absence of N-acetylglucosamine in wall teichoic acids of Listeria monocytogenes modifies biofilm architecture and tolerance to rinsing and cleaning procedures
Source: PLoS One. 2018 Jan 10;13(1):e0190879. doi: 10.1371/journal.pone.0190879 (PMC5761963; doi:10.1371/journal.pone.0190879)
Supplement: S1 Table — (PDF) [file pone.0190879.s003.pdf]

Table S1.

Strains	Accession# *lmo2549*	Accession# *lmo2550*	Strains	Accession# *lmo2549*	Accession# *lmo2550*
A26 P3	LT716843	LT716936	B7681 P1	LT716794	LT716887
A3 P3m	LT716844	LT716937	CS5d	LT716795	LT716888
A31 P1	LT716845	LT716938	C3935 PS	LT716796	LT716889
B106 OFT9	LT716846	LT716939	C4049 OT	LT716797	LT716890
B136 P2F	LT716847	LT716940	C3248 OS	LT716798	LT716891
B674 P1	LT716848	LT716941	C5125 O	LT716799	LT716892
B7202 O1	LT716849	LT716942	C5583 APL1	LT716800	LT716893
B7482 O1	LT716850	LT716943	DSS 1130 BFA2	LT716801	LT716894
B82 P2	LT716851	LT716944	DSS765 BA3	LT716802	LT716895
C2806 O	LT716852	LT716945	B1166 O1	LT716803	LT716896
C3143 O1	LT716853	LT716946	B1169 P1	LT716804	LT716897
C3299 O1	LT716854	LT716947	B131 P2	LT716805	LT716898
C3615 PS	LT716855	LT716948	B7201 P2	LT716806	LT716899
C4579 OS	LT716856	LT716949	C1530 O	LT716807	LT716900
C4627 OS	LT716857	LT716950	C4839 P1	LT716808	LT716901
C5086 T1	LT716858	LT716951	C5067 PS	LT716809	LT716902
C5128 P	LT716859	LT716952	CP622 CS1	LT716810	LT716903
C5142 DNT	LT716860	LT716953	CS163 P1	LT716811	LT716904
C5316 DNT4	LT716861	LT716954	DSS728 CA1	LT716812	LT716905
C5391 OT	LT716862	LT716955	DSS843 CFA2	LT716813	LT716906
C5400 PT	LT716863	LT716956	A32 O3	LT716814	LT716907
C58 O1	LT716864	LT716957	CP614 CS2	LT716815	LT716908
C838 P1	LT716865	LT716958	CPL631 AS1	LT716816	LT716909
CL110 P2T48	LT716866	LT716959	C4283 PS	LT716817	LT716910
CL219 S2	LT716867	LT716960	CP627 BFS1	LT716818	LT716911
CL229 L1	LT716868	LT716961	DA1139.1 E	LT716819	LT716912
CL297 AS1	LT716869	LT716962	B7880 P1	LT716820	LT716913
CP520 APS1	LT716870	LT716963	B8005 P1	LT716821	LT716914
D125 DNT1	LT716871	LT716964	C5070 DNT1	LT716822	LT716915
D1355 PT1	LT716872	LT716965	C54 P1	LT716823	LT716916
D2022 PS2	LT716873	LT716966	C5551 OT	LT716824	LT716917
DA1103 p1	LT716874	LT716967	CS328 CS1	LT716825	LT716918
DA146 q	LT716875	LT716968	CS566 A1	LT716826	LT716919
DA1559 a2	LT716876	LT716969	DA1283 q2	LT716827	LT716920
DA169.2 a6	LT716877	LT716970	DA1617 q2	LT716828	LT716921
DA185 S	LT716878	LT716971	C1063 O1	LT716829	LT716922
DA209 q1	LT716879	LT716972	DA1421 ic2	LT716830	LT716923
DM B5	LT716880	LT716973	DA1477 i4	LT716831	LT716924

DPF 234 HG2	LT716881	LT716974	DA509 i1	LT716832	LT716925
DPF235 HG6	LT716882	LT716975	DM K17c	LT716833	LT716926
DSS794 AA1	LT716883	LT716976	D829 OS2	LT716834	LT716927
DSS835 CA1	LT716884	LT716977	DA1583.3 a3	LT716835	LT716928
DSS836 CS1	LT716885	LT716978	B7678 P1	LT716836	LT716929
EGD-e	LT716886	LT716979	C3232 O1	LT716837	LT716930
			C3572 OS	LT716838	LT716931
			C5068 PT1	LT716839	LT716932
			CS536 PL1	LT716840	LT716933
			CS504 CA1	LT716841	LT716934
			DA L1	LT716842	LT716935
